# Tinnitus and normal hearing: a study on the transient otoacoustic emissions suppression

**DOI:** 10.1016/S1808-8694(15)30660-1

**Published:** 2015-10-19

**Authors:** Luciene da Cruz Fernandes, Teresa Maria Momensohn dos Santos

**Affiliations:** 1 MSc, professor; 2 PhD, professor

**Keywords:** efferent pathways, acoustic stimulation, tinnitus

## Abstract

The workings of the auditory pathway of patients with tinnitus and normal hearing can be associated with an auditory efferent pathway dysfunction at the level of the superior olivary complex. Otoacoustic emission suppression with contralateral noise can represent an alternative to its evaluation.

**Aim:**

to investigate Transient Otoacoustic Emission suppression in normal hearing adults with and without tinnitus and to compare the two groups. Study design: cross-sectional contemporary cohort.

**Materials and Methods:**

we assessed 40 female individuals between 18 and 59 years of age, 20 without tinnitus and 20 with it. We studied the TOAE suppression with a contralateral white noise at 50 dBSPL.

**Results:**

TOAE amplitude was lower in the group with tinnitus. There was no difference between the groups with and without tinnitus in terms of TOAE suppression, except in the frequency of 1000 Hz in the left ear in the tinnitus group.

**Conclusion:**

the afferent system assessment may contribute to the topographic diagnosis of tinnitus; however, we still need further studies to establish the proper methodology and normative values to carry out these tests.

## INTRODUCTION

Various theories have been proposed to better understand how the auditory pathways function in people with normal hearing and patients with tinnitus, to thus offer additional resources to health care professionals and offer patients improved follow-up and rehabilitation possibilities. One of them presupposes that alterations in central levels may introduce and/or maintain tinnitus and that efferent pathway disorders, affecting more specifically the superior olivary complex (SOC), may be one of the etiologies of tinnitus in patients with normal hearing^1^.

The human cochlea is innervated by efferent fibers rising from the ipsilateral and contralateral SOC called olivocochlear bundle and is composed by two systems: the medial, innervating the outer hair cells (OHC); and the lateral, innervating the inner hair cells (IHC)^2^. These systems impact the modulation of cochlear activity both by exciting or inhibiting it, and their function can be measured through the suppression of otoacoustic emissions^3^.

In normal individuals OAEs can be suppressed through contralateral stimulation. Absence of suppression may occur in tinnitus patients, suggesting a possible connection with medial efferent system disorder^4^. Fifty percent of tinnitus patients and 100% of hyperacusis patients cannot suppress OAEs, supporting both this theory and the idea that both symptoms share the same pathophysiological basis^5^,^6^.

Many authors have used contralateral OAE suppression to observe associations between complaints of tinnitus and possible medial efferent system disorders^4^, ^5^, ^6^, ^7^, ^8^. However, Filha (2005)^9^ discussed the difficulty in comparing data from different papers due to their methodological differences.

It is thus relevant to look into otoacoustic emission suppression in individuals complaining of tinnitus to verify possible alterations in their efferent systems and to check whether this particular test is indeed effective in the assessment of patients with this condition.

## OBJECTIVE

This paper aims to investigate contralateral suppression of transient evoked otoacoustic emissions in adults with normal hearing with and without complaints of tinnitus and compare the findings for both groups.

## MATERIALS AND METHOD

This study was approved by the Research Ethics Committee of our institution under permit 0022/2006.

This study was carried out at a School Clinic of Speech and Hearing Therapy located in Lauro de Freitas and at a specialized private clinic located in Salvador, Brazil.

This is a contemporary cohort cross-sectional study. Forty female* right-handed subjects with normal hearing and ages ranging between 18 and 59 years were enrolled in the study. They were distributed into two groups of 20 subjects each: - case group: subjects with normal hearing complaining of continuous/intermittent, low/high, unilateral/bilateral tinnitus for at least one month seen at the Speech and Hearing Therapy Clinic; - control group: subjects with normal hearing and no complaints of tinnitus, paired for age. All subjects underwent audiological assessment with tone threshold audiometry tests, logoaudiometry, and measurements of acoustic impedance: tympanometry and contralateral acoustic reflex assessment at 500Hz, 1000Hz, and 2000Hz and transient evoked OAEs using non-linear clicks with regular pulses of 80 microseconds of 80dB and a window of 0 to 10ms. Analyzed frequencies are 1 kHz, 1.5 kHz, 2 kHz, 3 kHz, 4 kHz, separately and combined. We used a Madsen Capella TEOAE device connected to a Pentium II computer running on Windows 98 with software NOAH release 2.0.

The study included subjects with normal audiometry10, tympanometry type A11, no history of neurologic or psychiatric disease, no history of risk for hearing disorders such as ototoxic medication and noise.

After signing a Free Informed Consent Term, subjects underwent TEOAE tests with and without contralateral noise. White noise at 50 dBNPS was used in the tests, as provided by an Interacoustics middle ear analyzer model AZ7 with TDH49 earphones. Tests were done with the patients seated in a soundproof room. Subject 1 did the test first on the right ear, subject 2 on the left ear and so on and so forth; two test sessions were done for each ear: a first without noise and a second with contralateral noise. Two tests were done for each TEOAE session to validate the test for assessment. Therefore, for result analysis, we used the first sample both for data with and without contralateral noise in the calculation of all TEOAE results, as no statistically significant differences were seen between the two samples.

OAEs were deemed present when reproducibility was equal to or greater than 50% and the signal/noise ratio was of at least 3 dB in the frequency ranges analyzed separately and jointly, as specified in the equipment manual; contralateral suppression of OAEs was deemed positive when values were equal to or greater than zero12; suppression effect was analyzed for reductions of 0.2, 0.4 and 0.6 dB between the TEOAE amplitude with and without contralateral noise, i.e., we looked at which of these values would provide for positive suppression effect to better characterize the groups, given that there is some discrepancy in the literature as to the minimum value to be accounted for given the wide variation in the method used in other studies^9^.

Results were interpreted using descriptive values, Wilcoxon test and Mann-Whitney non-parametric test. The significance level was set at 5% (? = 0.05).

## RESULTS

The mean age of the tinnitus-free group was 45.56 years (standard deviation of 9.67 years), while the tinnitus group had a mean age of 45.62 years (standard deviation of 9.48 years).

**Graphs 1 and 2:**
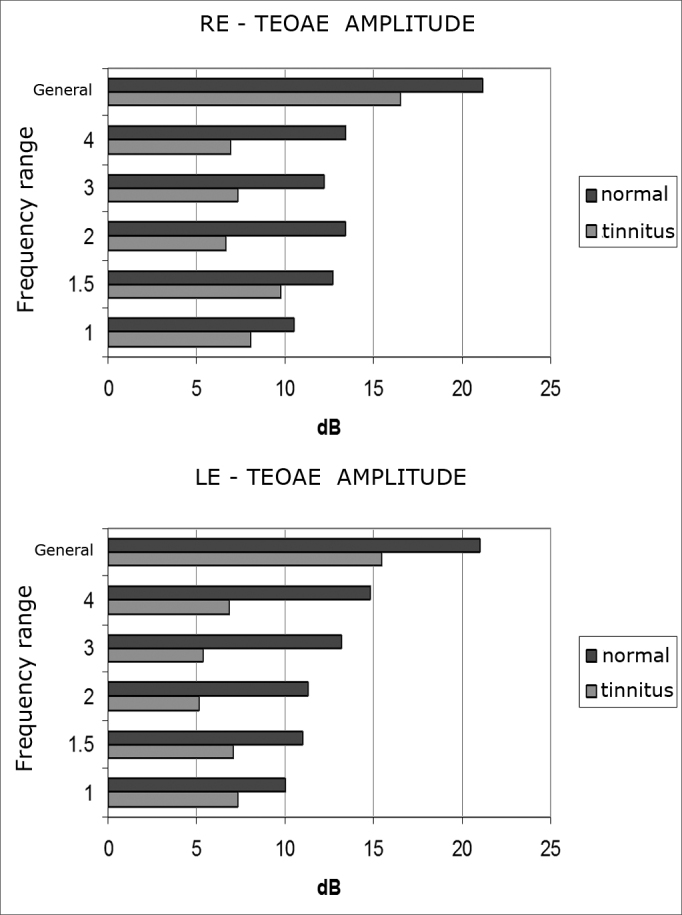
Mean TEOAE amplitude without contralateral noise per frequency range and in general for right and left ears in the tinnitus (n= 20) and tinnitus-free (n= 20) groups.

Fifteen percent of the patients in the tinnitus group complained of right ear tinnitus, 65% of left-ear tinnitus, and 20% of bilateral tinnitus. Forty-five percent of them complained of low pitch tinnitus, and 55% of high-pitch tinnitus. (Graphs 1 and 2)

**Graphs 3 and 4:**
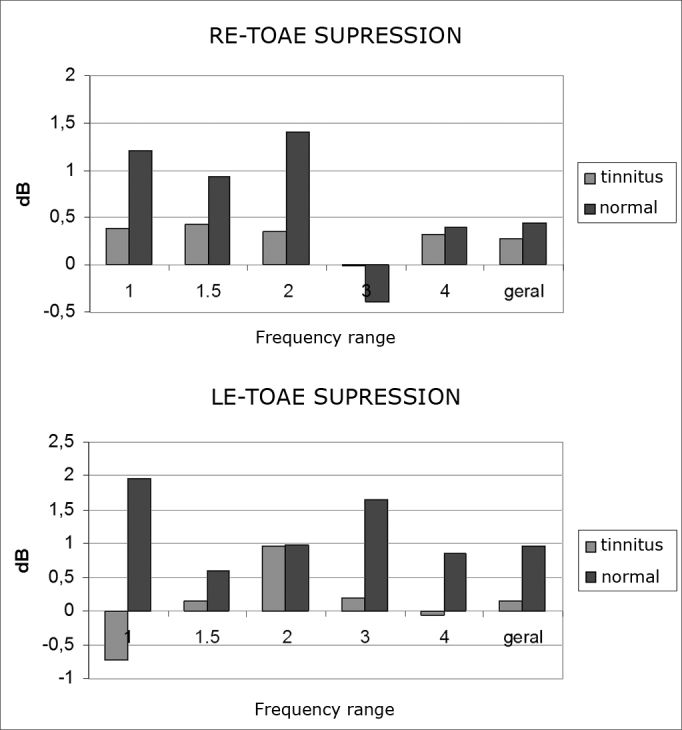
Mean TEOAE amplitude suppression per frequency range for right and left ears in the tinnitus (n= 20) and tinnitus-free (n= 20) groups.

Mean TEOAE amplitude values in all frequencies were lower in the left ears of both groups. The tinnitus group had lower amplitude values for all frequencies in both right and left ears (Graphs 3 and 4).

There was no significant difference between groups when we looked at their right ears, while significant differences were found only in left ears at 1 kHz (Chart 1).

**Chart 1:** Descriptive levels (p-values) from the comparison of suppression outcomes per frequency range, separately and combined, between right and left ears.

Frequency (kHz)	Tinnitus Group	Tinnitus-free Group
1	0,0347 *	0,2673
1,5	0,2972	0,8497
2	0,7147	0,8497
3	0,6451	0,0785
4	0,5788	0,7250
Combined	0,2776	0,3430

* p-value (Mann Whitney Test) = 0.0347

Statistically significant differences were only observed at the 1 kHz frequency (Table 1).

**Table 1:** Percentages of subjects with present or absent TEOAE suppression at 0.2, 0.4 or 0.6 dB as the minimum suppression levels in the given frequencies.

Freq.	Groups^1^	Suppression					
		0,2		0,4		0,6	
		Present	Absent	Present	Absent	Present	Absent
1,0	G1	13 (44,8%)	7 (63,6)	11 (42,3)	9 (64,3)	11 (42,3)	9 (64,3)
	G2	16 (55,2%)	4 (36,4)	15 (57,7)	5 (35,7)	15 (57,7)	5 (35,7)
P- value 2	0,2881	0,1848	0,1848				
1,5	G1	15 (50,0)	5 (50,0)	14 (48,3)	6 (54,6)	13 (52,0)	7 (46,7)
	G2	15 (50,0)	5 (50,0)	15 (51,7)	5 (45,5)	12 (48,0)	8 (53,3)
P- value 2	1,0000	0,7233	0,7440				
2,0	G1	15 (53,6)	5 (41,7)	15 (53,6)	5 (41,7)	13 (50,0)	7 (50,0)
	G2	13 (46,4)	7 (58,3)	13 (46,4)	7 (58,3)	13 (50,0)	7 (50,0)
P- value 2	0,4902	0,4902	1,0000				
3,0	G1	15 (51,7)	5 (45,5)	13 (48,2)	7 (53,8)	10 (43,5)	10 (58,8)
	G2	14 (48,3)	6 (54,6)	14 (51,8)	6 (46,2)	13 (56,5)	7 (41,2)
P- value 2	0,7233	0,7357	0,3373				
4,0	G1	14 (48,3)	6 (54,6)	12 (44,4)	8 (61,5)	10 (41,7)	10 (62,5)
	G2	15 (51,7)	5 (45,5)	15 (55,6)	5 (38,5)	14 (58,3)	6 (37,5)
P- value 2	0,7233	0,3112	0,1967				

* Only one male patient from the group selected for the study agreed to participate, and we thus decided to keep only female subjects.

1 G1 = Tinnitus group; G2 = Tinnitus-free group.

2 Descriptive levels (p-value) derived from Pearson's chi-square test.

**Graphs 5 and 6:**
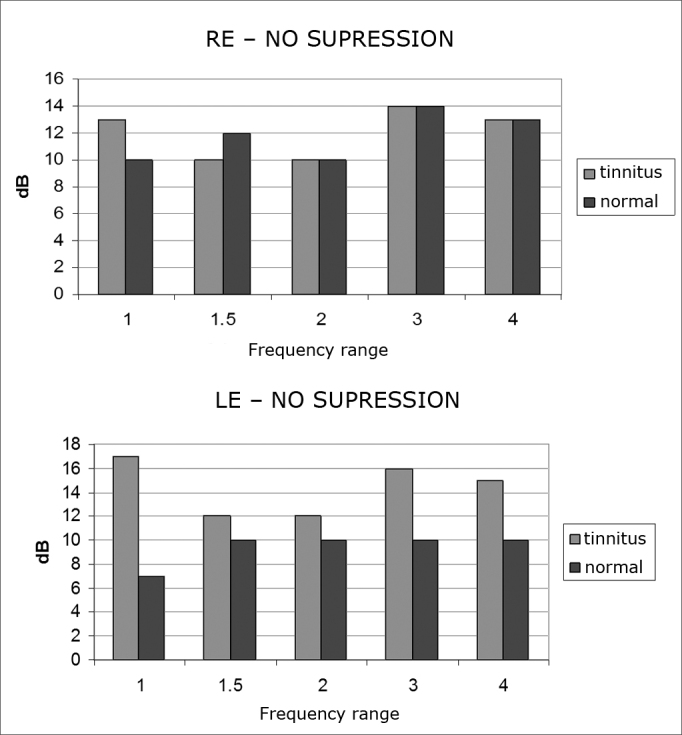
Number of subjects not presenting TEOAE suppression at 0.6dB of minimum level required for suppression effect per frequency range and ear side in the tinnitus (n= 20) and tinnitus-free (n= 20) groups.

There was no statistically significant difference between values of 0.2, 0.4, and 0.6dB as the minimum levels to verify the presence of a suppression effect. At 0.6dB, however, the p-value was lower for most frequencies. This value was then picked in the other tables as the minimum value in the consideration of the suppression effect (Graphs 5 and 6).

In the tinnitus group, only left ears in frequencies of 1 and 3 kHz presented a statistically significant difference.

## DISCUSSION

Tinnitus was predominantly observed unilaterally in the left ear, followed by bilateral tinnitus, and right ear unilateral tinnitus. Such finding is in agreement with reports from Lee et al.^13^ as most of the subjects in their study also had left ear unilateral tinnitus. However, Barros et al.^14^ reported on a case group in which most subjects complained of bilateral tinnitus. Therefore, tinnitus may have unilateral and bilateral manifestations.

Most subjects complained of tinnitus that started off as a high pitch noise, followed by what they perceived as a low pitch noise, as also reported by Lee et al.^13^.

Mean TEOAE amplitude values were lower in the tinnitus group when compared to the tinnitus-free group on all analyzed frequencies and for both left and right ears. No differences were found in the standard deviation of either of the groups. These findings are in agreement with reports by Jastreboff and Hazell^15^. They found that the first stage called tinnitus generation occurs usually in the peripheral auditory system (cochlea and auditory nerve). Therefore, the fact that the TEOAEs had lower amplitudes in the tinnitus group may be related to peripheral hearing disorders and, even though they are not measured through conventional audiometry, may have contributed to the onset of tinnitus.

In relation to TEOAE contralateral suppression in the various frequencies and in general, there was no statistically significant difference between the case and control groups for the right ear. In left ears, however, statistically significant difference was found at 1000 Hz. These findings are indicative of MOC system disorder in tinnitus patients^5^,^8^. The same was however not observed by Chèry-Croze et al.^6^ and Filha^9^, as they did not find statistically significant differences in TEOAE suppression in the tinnitus groups.

When comparing right and left ears in terms of TEOAE contralateral suppression in each of the frequencies and in all combined, statistically significant difference was found only at 1000 Hz on the left ears of the tinnitus group. This finding is supported by Khalfa et al.^16^, as they reported increased effectiveness of the MOC system in the right ear of right-handed subjects free of hearing disorders; their group had only two females who, according to the authors, had a less striking asymmetry than males.

The relationship between MOC system asymmetry, hemisphere laterality, and gender requires further clarification in order for tinnitus to be clearly connected with MOC disorders.

There is great difficulty in categorizing TEOAE contralateral suppression in terms of presence or absence of suppression, as methods vary quite substantially, impairing even further the possibilities of establishing comparisons between papers available in the medical literature^9^. This is the reason why we decided, in this study, to check if there are differences between considering 0.2, 0.4 or 0.6 dB as minimum values to produce suppression. No statistically significant differences were observed between these values, although at 0.6 dB the p-value was lower in most frequencies. Thus, we opted to consider 0.6 dB as the minimum value for suppression effect presence assessment.

In relation to the suppressive effect between the ears in the frequencies of 1.0, 1.5, 2.0, 3.0 e 4.0 kHz, we saw that only left ears at 1 and 3 kHz from the tinnitus group presented statistically significant differences. Such difference in the left ears of the tinnitus group may suggest disorders at the level of the superior olivary complex, a system that participates in TEOAE suppression^17^. The results described above show possible involvement of the MOC system in tinnitus patients and speak of the relevance of OAEs in the topodiagnostic assessment of these subjects^1^,^5^,^6^,^8^.

TEOAE contralateral suppression presented statistically significant differences in the comparison of tinnitus and tinnitus-free groups. Nonetheless, there is still no consensus for the definition of cases in which the efferent system may be related to tinnitus. Therefore, additional studies on the pathophysiology of tinnitus and more method standardization concerning values for TEOAE suppression are required.

## CONCLUSIONS

Transient evoked otoacoustic emission amplitude is lower in all frequency ranges and in both ears of subjects with tinnitus; suppression of transient evoked otoacoustic emissions are more dramatic in the ear subjects report to be affected by tinnitus.
